# ‘Most people still believe that kuru is caused by sorcery’

**DOI:** 10.1098/rstb.2008.4025

**Published:** 2008-11-27

**Authors:** Anderson Puwa

**Affiliations:** Papua New Guinea Institute of Medical ResearchPO Box 60, Goroka, EHP 441, Papua New Guinea

This is the second time I have come to England to the MRC Prion Unit. I have been working for the Kuru Research Project in the Papua New Guinea Institute of Medical Research for 6 years. Now, I am the Community Liaison Officer. I am also the community leader of Waisa Village. In our kuru studies, I work closely with Jerome Whitfield and Michael Alpers.

In the early 1960s, when I first saw Michael Alpers in Waisa, I was 7 years old. Michael has always been a good friend of our family. The kuru research work went ahead successfully. Later Michael became the Director of the Papua New Guinea Institute of Medical Research. The Institute studies all the sicknesses of Papua New Guinea and we are pleased to have Prof. Peter Siba now as its Director.

My late father Mr Puwa helped Michael with his work and supported him personally. He assisted with the examination of patients and the collection of samples from patients and others. He explained to the family and community why these samples of blood, brain and the like were needed. Working together, my father and Michael made a film on traditional salt making, a skill and technology that has now died out, since my father was the acknowledged salt maker of the village.

Although most people still believe that kuru is caused by sorcery, there are a few of us who understand how it came to our people. I am very happy about all the research work for the last 50 years that has given us this understanding.

